# Molecular Dynamics of Jelly Candies by Means of Nuclear Magnetic Resonance Relaxometry

**DOI:** 10.3390/molecules28052230

**Published:** 2023-02-27

**Authors:** Danuta Kruk, Leonid Grunin, Aleksandra Stankiewicz, Karol Kołodziejski, Esmanur Ilhan, Mecit Halil Oztop

**Affiliations:** 1 Department of Physics & Biophysics, University of Warmia and in Olsztyn, 10-719 Olsztyn, Poland; 2 Resonance Systems GmbH, 73230 Kirchheim unter Teck, Germany; 3 Department of Food Engineering, Middle East Technical University, Ankara 06800, Turkey

**Keywords:** nuclear magnetic resonance, relaxation, dynamics, jelly candies

## Abstract

^1^H spin-lattice Nuclear Magnetic Resonance relaxation studies have been performed for different kinds of Haribo jelly and Vidal jelly in a very broad frequency range from about 10 kHz to 10 MHz to obtain insight into the dynamic and structural properties of jelly candies on the molecular level. This extensive data set has been thoroughly analyzed revealing three dynamic processes, referred to as slow, intermediate and fast dynamics occurring on the timescale of 10^−6^ s, 10^−7^ s and 10^−8^ s, respectively. The parameters have been compared for different kinds of jelly for the purpose of revealing their characteristic dynamic and structural properties as well as to enquire into how increasing temperature affects these properties. It has been shown that dynamic processes in different kinds of Haribo jelly are similar (this can be treated as a sign of their quality and authenticity) and that the fraction of confined water molecules is reduced with increasing temperature. Two groups of Vidal jelly have been identified. For the first one, the parameters (dipolar relaxation constants and correlation times) match those for Haribo jelly. For the second group including cherry jelly, considerable differences in the parameters characterizing their dynamic properties have been revealed.

## 1. Introduction

Nuclear Magnetic Resonance (NMR) relaxometry (relaxation experiments performed versus frequency) is a broadly appreciated method providing deep insight into the dynamic properties of molecular and ionic systems of various complexity, from liquids [1,2,3,4,5,6,7], via macromolecular systems such as polymers or proteins [8,9,10,11,12] to complex solids [13,14,15]. Classical NMR experiments are performed at a single magnetic field (resonance frequency) while, thanks to Fast Field Cycling technology [16,17], in NMR relaxometry experiments the resonance frequency can be varied over several orders of magnitudes (typically from about 10 kHz to tenths of MHz, referring to ^1^H resonance frequency). Consequently, one can probe in a single experiment into dynamic processes at very different time scales. In the case of ^1^H relaxation processes, the predominating relaxation mechanism is provided by ^1^H-^1^H mutual dipole-dipole interactions that fluctuate in time as a result of molecular motion (such as translational diffusion, molecular tumbling or the internal dynamics of molecules and ions). A sample containing hydrogen atoms gains a magnetization (magnetic moment) when placed in an external magnetic field. ^1^H nuclei possess magnetic moments that can be oriented parallel or antiparallel to the external magnetic field. The two states are associated with different energy. The energy difference between the two states (being proportional to the applied magnetic field) expressed in frequency units is referred to as the resonance frequency. According to the Boltzmann distribution, the lower energy state is preferred. Consequently, as a result of the difference between the number of ^1^H nuclei with magnetic moments oriented in parallel and antiparallel to the magnetic field, the sample gains magnetization. When the magnetic field changes, for instance, reduced to a lower value, the difference in the populations of the two energy levels, and hence the magnetization, becomes smaller. This implies that the magnetization evolves in time (usually in an exponential way) from the initial value to the new equilibrium. The time constant characterizing the evolution of the magnetization is referred to as spin-lattice relaxation time (its reciprocal value is called relaxation rate). The value of the relaxation rate depends on the strength of the dipole–dipole interactions, the time scale of their fluctuations and the mechanism of the motion. In this way, one extracts information on molecular motion from NMR relaxometry data.

Even this brief outline gives the clear impression that the price to pay for the unique information provided by NMR relaxometry is advanced data analysis. In our opinion, this is the main reason why NMR relaxometry is rarely exploited in food science. However, at this stage one should point out that in recent years the method has also rapidly gained popularity in this area. In fact, one can exploit NMR relaxometry in food science in two ways. The first is less challenging, but not less useful. One can treat the ‘relaxation dispersion profiles’ (frequency dependencies of spin-lattice relaxation rates) as a fingerprint of the authenticity and quality of specific food products. In this case, the analysis is limited to a qualitative assessment of the level of agreement between relaxation data for a specific sample and to a ‘model curve’, i.e., an averaged result of relaxation data for many samples of confirmed origin and quality. This approach has been, for instance, applied to differentiate between balsamic vinegars [18]. One can also, however, go further and perform a quantitative analysis of NMR relaxometry data for food products using relaxation models derived for complex molecular systems (food products can definitely be treated as such). Such an analysis enables the obtaining of a series of specific parameters characterizing the molecular dynamics and structure of food products, such as translation diffusion coefficients or dipolar relaxation constants. Examples of this category of studies are [19,20,21], dealing with dynamic properties of oil, honey [22] or water dynamics in eggs [23], cheese [24] and some other food systems [25,26]. This kind of approach reveals the dynamic and structural properties of food products at the molecular level.

The present work combines both approaches for jelly candies. Jelly candies are the most popular confectionery products. They are mostly produced by mixing a gelling agent, usually a polymer, with sugar, corn syrup, coloring and flavoring agents [27,28]. The gelling agent can be gelatin [27,29,30], pectin [31,32] or starch [33,34]. In this work, NMR relaxometry is exploited to reveal and quantitatively describe the dynamic properties of jelly candies as well as to explore the potential of this method for authentication purposes.

## 2. Theory

^1^H relaxation processes are caused by ^1^H-^1^H magnetic dipole–dipole interactions that fluctuate in time as a result of molecular motion. As pointed out in the Introduction, the Fast Field Cycling technology enables the performance of relaxation experiments over a broad range of magnetic fields (resonance frequencies), from below 10 kHz to 10 MHz in this case. This implies that in such experiments one can probe dynamic processes occurring at a time scale from milliseconds to nanoseconds. To obtain insight into the molecular dynamics, one can decompose the relaxation data into components associated with dynamic processes occurring on long, intermediate and fast time scales. According to relaxation theory [35,36,37], the ^1^H spin-lattice relaxation rate, $R_{1}\left(\omega \right)$ (ω denotes the ^1^H resonance frequency in angular frequency units), can be expressed as:

(1) $$R_{1}\left(\omega \right)=C^{DD}\left[\frac{\tau_{c}}{1+{\left(\omega \tau_{c}\right)}^{2}}+\frac{4\tau_{c}}{1+{\left(2\omega \tau_{c}\right)}^{2}}\right]$$

where $C^{DD}$ is referred to as a dipolar relaxation constant (dependent on the structure of the system), while $\tau_{c}$ denotes a correlation time, a characteristic time constant describing the time scale of the molecular motion. Anticipating the results, the relaxation data for jelly products can be reproduced in terms of three relaxation contributions associated with dynamic processes referred to as slow, intermediate and fast. Consequently, the relaxation rate can be expressed as [11,13]:

(2) $$R_{1}\left(\omega \right)=C_{s}^{DD}\left[\frac{\tau_{s}}{1+{\left(\omega \tau_{s}\right)}^{2}}+\frac{4\tau_{s}}{1+{\left(2\omega \tau_{s}\right)}^{2}}\right]+C_{i}^{DD}\left[\frac{\tau_{i}}{1+{\left(\omega \tau_{i}\right)}^{2}}+\frac{4\tau_{i}}{1+{\left(2\omega \tau_{i}\right)}^{2}}\right]\;+C_{f}^{DD}\left[\frac{\tau_{f}}{1+{\left(\omega \tau_{f}\right)}^{2}}+\frac{4\tau_{f}}{1+{\left(2\omega \tau_{f}\right)}^{2}}\right]+A$$

where $\tau_{s},\;\tau_{i}$ and $\tau_{f}$ denote the correlation times characterizing the slow, intermediate and fast dynamics, respectively, while $C_{s}^{DD},\;C_{i}^{DD}$ and $C_{f}^{DD}$ are the corresponding dipolar relaxation constants. The parameter A denotes a frequency independent term. The frequency independent term represents a relaxation process associated with a motion occurring at a time scale of the order of tenths of nanoseconds or faster, for instance, the internal dynamics of macromolecules.

## 3. Results and Analysis

**Figure 1:**
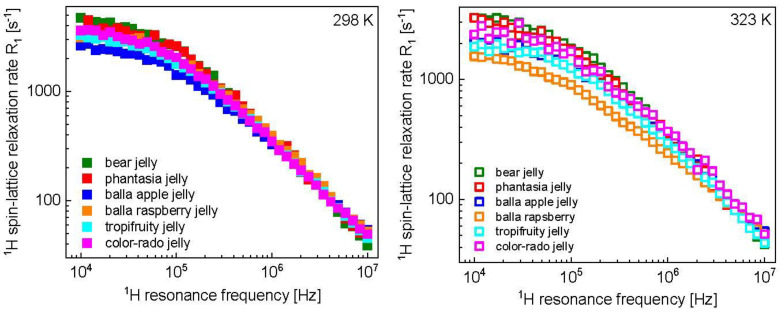
^1^H spin-lattice relaxation rates for various kinds of Haribo jelly at 298 K (**left**) and 323 K (**right**).

The ^1^H spin-lattice relaxation data for various kinds of Haribo jelly are shown in Figure 1.

**Figure 2:**
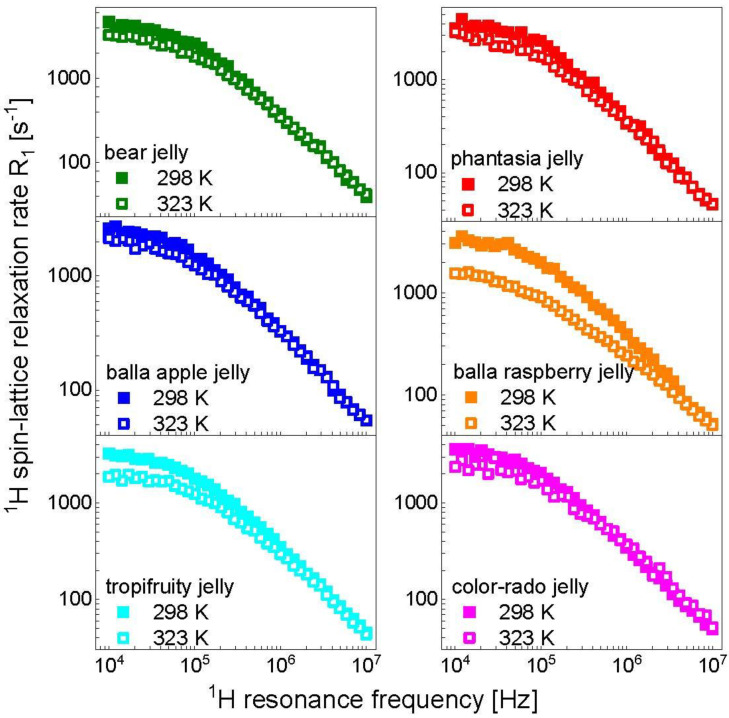
Comparison of ^1^H spin-lattice relaxation rates for different kinds of Haribo jelly at 298 K and 323 K.

The differences in relaxation rates manifest themselves mostly in the low frequency range. As far as the influence of temperature is concerned, Figure 2 shows a comparison of the relaxation data at 298 K and 323 K for the individual kinds of Haribo jelly. It can immediately be seen that the extent to each the data are affected by temperature variations for different kinds of the jelly. 

**Figure 3:**
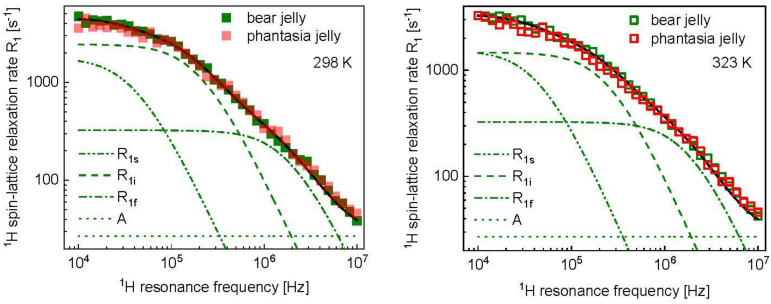
^1^H spin-lattice relaxation data for Haribo bear jelly and Haribo phantasia jelly at 298 K (**left**) and 323 K (**right**) reproduced in terms of the model described in the theory. The fit ($R_{1}$) shown as a black solid line has been decomposed into relaxation contributions associated with slow, intermediate and fast dynamics, $R_{1s}$, $R_{1i}$ and $R_{1f}$, respectively. The frequency independent term, A, is also indicated.

To obtain a quantitative insight into the dynamics, the data have been analyzed in terms of the model described in the theory. The fits for bear jelly are shown in Figure 3. The overall relaxation rate, $R_{1}$, has been decomposed into relaxation contributions associated with slow, intermediate and fast dynamics, $R_{1s}$, $R_{1i}$ and $R_{1f}$, respectively. As the data for Haribo bear jelly and Haribo phantasia jelly almost overlap at both temperatures, the same parameters have been attributed to both kinds of jelly (Figure 3 and Table 1).

**Figure 4:**
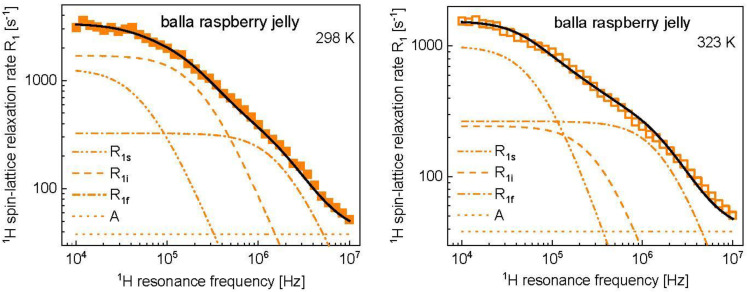
^1^H spin-lattice relaxation data for Haribo raspberry jelly at 298 K (**left**) and 323 K (**right**) reproduced in terms of the model described in the theory. The fit ($R_{1}$) shown as a black solid line has been decomposed into relaxation contributions associated with slow, intermediate and fast dynamics, $R_{1s}$, $R_{1i}$ and $R_{1f}$, respectively. The frequency independent term, A, is also indicated.

**Figure A1:**
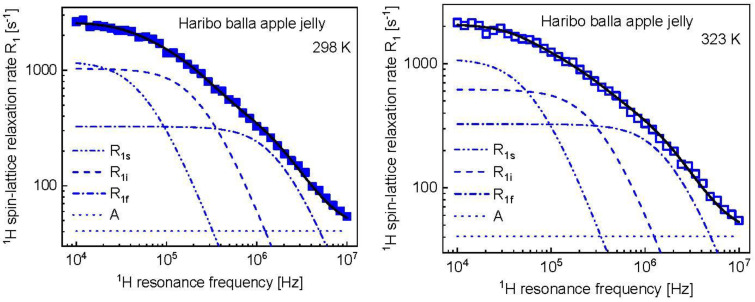
^1^H spin-lattice relaxation data for Haribo bara apply jelly at 298 K (**left**) and 323 K (**right**) reproduced in terms of the model described in the theory. The fit ($R_{1}$) shown as a black solid line has been decomposed into relaxation contributions associated with slow, intermediate and fast dynamics, $R_{1s}$, $R_{1i}$ and $R_{1f}$, respectively. The frequency independent term, A, is also indicated.

**Figure A2:**
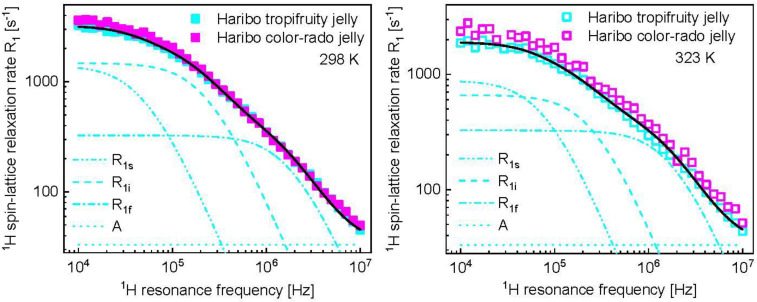
^1^H spin-lattice relaxation data for Haribo tropifruity jelly and Haribo color-rado jelly at 298 K (**left**) and 323 K (**right**) reproduced in terms of the model described in the theory. The fit ($R_{1}$) shown as a black solid line has been decomposed into relaxation contributions associated with slow, intermediate and fast dynamics, $R_{1s}$, $R_{1i}$ and $R_{1f}$, respectively. The frequency independent term, A, is also indicated.

**Figure A3:**
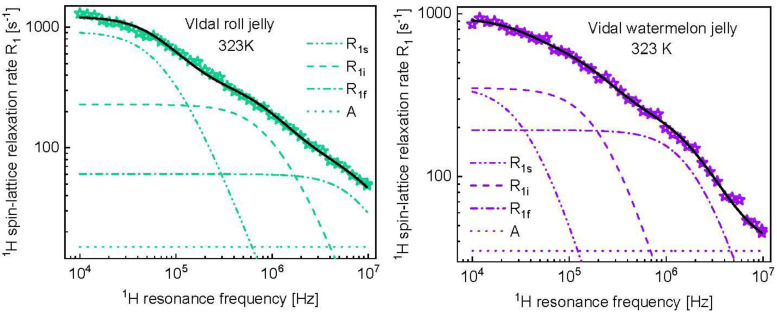
^1^H spin-lattice relaxation data for Vidal roll jelly (**right**) and Vidal watermelon jelly (**left**) at 323 K reproduced in terms of the model described in the theory. The fit ($R_{1}$) shown as a black solid line has been decomposed into relaxation contributions associated with slow, intermediate and fast dynamics, $R_{1s}$, $R_{1i}$ and $R_{1f}$, respectively. The frequency independent term, *A*, is also indicated.

The strategy of the analysis relies on explaining the differences in the relaxation rates at 298 K and 323 K in terms of as few parameters as possible. It has turned out that, for Haribo bear jelly and Haribo phantasia jelly, the differences can be attributed to changes in the parameters characterizing the intermediate time scale dynamics, $C_{i}^{DD}$ and $\tau_{i}$. The same strategy has been applied to the analysis of the relaxation data for other kinds of Haribo jelly. The obtained parameters are collected in Table 1**.** For balla apple jelly, the temperature differences are also small but, to reproduce the relaxation data changes in the correlation time, characterization of the slow dynamics are required, in addition to the parameters describing the dynamic process occurring on the intermediate scale. The fits are shown in Appendix A (Figure A1, Figure A2 and Figure A3). The temperature effect for balla raspberry jelly is significant. The relaxation scenario is shown in Figure 4.

As, in this case, the temperature also affects the relaxation data at high frequency, to reproduce the result, changes in the dipolar relaxation constant associated with the fast dynamics, $C_{f}^{DD}$, were needed; however, the parameter changed only slightly. The differences between the relaxation data at 298 K and 323 K primarily stem from the intermediate dynamic process (Figure 4, Table 1), although the correlation time of the slow dynamics, $\tau_{s}$, has also become somewhat shorter. Following the line, the relaxation data for tropifruity jelly and color-rado jelly at 298 K show only small deviations and, consequently, they have been reproduced in terms of the same parameter included in Table 1. The corresponding figure, showing the fit and its decomposition, has been included in Appendix A. The same applies to the jelly at 323 K—the temperature effect is very similar and can be, in a good approximation, described by the same set of parameters given in Table 1. (The corresponding figure has been included in Appendix A).

**Figure 5:**
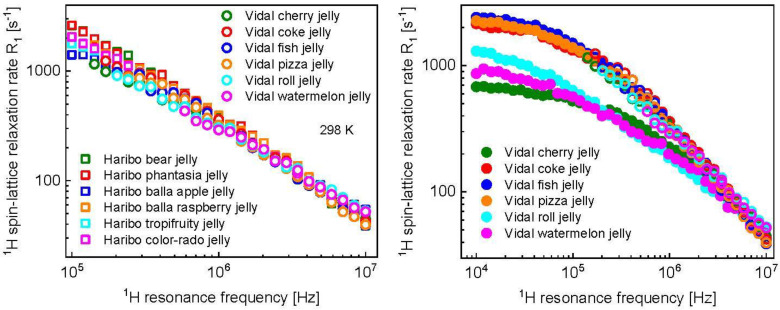
(**left**) ^1^H spin-lattice relaxation rates for various kinds of Vidal jelly at 298 K compared with ^1^H spin-lattice relaxation rates for various kinds of Haribo jelly at 298 K, (**right**) ^1^H spin-lattice relaxation rates for various kinds of Vidal jelly at 298 K (open symbols) and 323 K (solid symbols).

For comparison, ^1^H spin-lattice relaxometry experiments have been performed for Vidal jelly at 298 K and 323 K. It occurred that, at 298 K, the experiment could be performed only at high and intermediate frequencies. It can be stated that, in that range, the relaxation features of Vidal jelly follow those of Haribo jelly (Figure 5). At the same time, one should realize that at low frequencies the relaxation processes in Vidal jelly are very fast; this is the reason for not being able to measure the relaxation rates as they exceed the limit of the relaxation rates accessible for FFC-NMR relaxometry. As the relaxation processes become slower with increasing temperature, at 323 K the relaxation data have been obtained for the whole frequency range (Figure 5).

**Figure 6:**
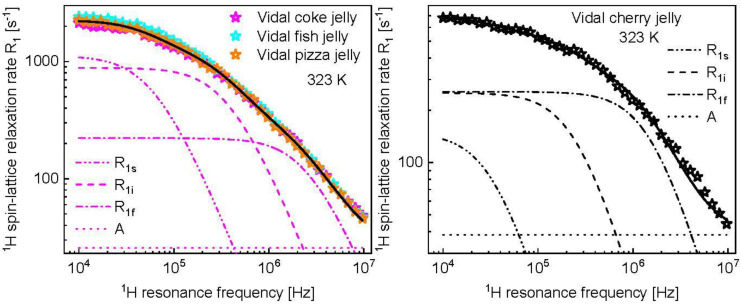
^1^H spin-lattice relaxation data for Vidal coke jelly, Vidal fish jelly and Vidal pizza jelly at 323 K (**left**) and Vidal cherry jelly at 323 K (**right**) reproduced in terms of the model described in the theory. The fit ($R_{1}$) shown as a black solid line has been decomposed into relaxation contributions associated with slow, intermediate and fast dynamics, $R_{1s}$, $R_{1i}$ and $R_{1f}$, respectively. The frequency independent term, A, is also indicated.

The data have been analyzed with a full analogy to the data for Haribo jelly. As the data for coke, fish and pizza jelly overlap, they have been reproduced in terms of one set of parameters (Figure 6) collected in Table 2. It is of interest to note that the dipolar relaxation constant, $C_{s}^{DD}$ remained unchanged compared to Haribo jelly. Figure 6 also shows the case of Vidal cherry jelly as a further example, while the analysis for Vidal roll jelly and Vidal watermelon jelly is shown in Appendix A. The parameters for all kinds of jelly are collected in Table 2. The results are discussed in the next section.

## 4. Discussion

^1^H spin-lattice relaxation data for different kinds of Haribo jelly, collected at 298 K (Figure 1, left), show some differences in the low frequency range; in the high frequency range, the relaxation rates become very similar. These features are reflected by the parameters obtained from the data analysis. The correlation time and the dipolar relaxation constant characterizing the fast dynamic processes dominating the relaxation in the high frequency range, $\tau_{f}$ and $C_{f}^{DD}$, are the same for all kinds of Haribo: $\tau_{f}$ = 5.21 × 10^−8^ s and $C_{f}^{DD}$ = 1.25 × 10^9^ Hz^2^. As far as the dynamics at an intermediate timescale is concerned, the correlation time varies in a narrow range, from $\tau_{i}$ = 3.97 × 10^−7^ s for balla apple jelly to $\tau_{i}$ = 4.83 × 10^−7^ s for bear jelly and phantasia jelly. The corresponding dipolar relaxation constant varies from $C_{i}^{DD}$ = 5.20 × 10^8^ Hz^2^ for balla apple jelly to $C_{i}^{DD}$ = 1.00 × 10^9^ Hz^2^ (approximately twice as large) for bear jelly and phantasia jelly. The dipolar relaxation constant associated with the slow dynamic process is the same for all kinds of Haribo jelly, $C_{s}^{DD}$ = 1.46 × 10^8^ Hz^2^, while the correlation time varies between $\tau_{s}$ = 1.92 × 10^−6^ s for tropifruity jelly and color-rado jelly to $\tau_{s}$ = 2.43 × 10^−6^ s for bear jelly and phantasia jelly. The slow dynamics can be attributed to the motion of the macromolecular fraction and water molecules attached to the network for a time longer than the obtained correlation time. Figure 5 shows that the temperature effect depends on the kind of jelly—the most pronounced changes in the relaxation rates are observed for balla raspberry jelly, while the relaxation rates for bear jelly and balla apple jelly are only slightly affected by increasing temperature. One can immediately see from Table 1 that the fast dynamic process remains, in fact, unaffected by temperature—only for balla raspberry jelly does the dipolar relaxation constant become somewhat lower ($C_{f}^{DD}$ = 1.02 × 10^9^ Hz^2^ instead of 1.25 × 10^9^ Hz^2^), but still within the fitting uncertainty. The dipolar relaxation constant for the slow dynamics, $C_{s}^{DD}$, remains unchanged, while the corresponding correlation time, $\tau_{s}$, shows, overall, only slight changes: for bear jelly and phantasia jelly the correlation time remains unchanged, while for tropifruity jelly and color-rado jelly the most significant changes are observed, yet the ratio between the correlation times at 298 K and 323 K is 1.6. Consequently, the analysis shows that the relaxation rates mostly reflect changes in the intermediate dynamics that are affected by temperature. Moreover, increasing temperature leads to decreasing of the dipolar relaxation constant, $C_{i}^{DD}$. The relaxation constant is especially reduced for balla raspberry jelly (by a factor of 4.4), while the correlation time, $\tau_{i}$, is affected to a much lesser extent. Although this cannot be proved, we suppose that the intermediate dynamic process represents the dynamics of water molecules confined in the macromolecular matrix. The presumption is based on comparison with the order of relaxation rates attributed to the motion of confined water molecules in other food products [23,24,25,26]. In such a case, the dipolar relaxation constant, $C_{i}^{DD}$, reflects (is proportional to) the mole fraction of water molecules in the bound position [38] which becomes lower with increasing temperature.

Following this line, it has turned out that ^1^H spin-lattice relaxation rates for different kinds of Vidal jelly do not differ much in the frequency range from about 0.1 MHz to 10 MHz (Figure 5, left). However, at lower frequencies the relaxation processes became too fast to perform the measurements for all kinds of Vidal jelly. The reason for this can be long correlation times $\tau_{s}$ or/and larger (compared to Haribo jelly) dipolar relaxation constants, $C_{s}^{DD}$. As with increasing temperature the relaxation process becomes slower, at 323 K the relaxation rates have been measured in the whole frequency range (Figure 5, right). The data form two distinct groups. The first group includes the data for coke jelly, fish jelly and pizza jelly—the data almost coincide and do not differ (in the frequency range from about 0.1 MHz to 10 MHz) from those at 298 K. The second group includes data for cherry jelly, watermelon jelly and roll jelly—the data clearly show temperature effects and differ among themselves in the low frequency range. As far as the first group is concerned, the dipolar relaxation constant for the slow dynamic process (that presumably corresponds to the dynamics of the macromolecular fraction), $C_{s}^{DD}$ = 1.46 × 10^8^ Hz^2^, is the same as for Haribo jelly. Analogously, the correlation time, $\tau_{s}$ = 1.53 × 10^−6^ s, is in the range of the corresponding correlation times for Haribo jelly (from 1.20 × 10^−6^ s to 2.43 × 10^−6^ s). The dipolar relaxation constant associated with the intermediate dynamics, $C_{i}^{DD}$ = 6.98 × 10^8^ Hz^2^, is close to the value obtained for Haribo bear jelly and Haribo phantasia jelly, while the correlation time, $\tau_{i}$ = 2.53 × 10^−7^ s, is close to that for Haribo balla raspberry jelly. Eventually, the dipolar relaxation constant $C_{f}^{DD}$ = 1.25 × 10^9^ Hz^2^ is equal to that for Haribo jelly and the correlation time $\tau_{f}$ = 3.55 × 10^−8^ s is comparable to that for Haribo jelly (5.21 × 10^−8^ s). This implies that, at 323 K, the dynamic and structural properties of Vidal coke jelly, fish jelly and pizza jelly are similar to that of Haribo jelly. The situation is different for Vidal cherry jelly. The essential difference is in the dipolar relaxation constant, $C_{s}^{DD}$ = 1.16 × 10^7^ Hz^2^, more than 10 times lower than that for Haribo jelly and the coke, fish and pizza Vidal jelly group. The correlation time, $\tau_{s}$, is close to that for Haribo bear jelly and Haribo phantasia jelly. The dipolar relaxation constant for the intermediate dynamics is low, $C_{i}^{DD}$ = 1.38 × 10^8^ Hz^2^ (the lowest for Haribo jelly yields 1.78 × 10^8^ Hz^2^ for balla raspberry jelly), but the corresponding correlation time, $\tau_{i}$, matches that for Haribo jelly. The dipolar relaxation constant for the fast dynamics is somewhat lower, while the corresponding correlation time is somewhat longer than for Haribo jelly and the coke, fish and pizza Vidal jelly group. For Vidal roll jelly the parameters characterizing the slow dynamics correspond to that for Haribo jelly and the coke, fish and pizza Vidal jelly group ($C_{s}^{DD}$ = 1.46 × 10^8^ Hz^2^); the same applies to the dipolar relaxation constant for the intermediate dynamics, $C_{i}^{DD}$. However, for Vidal roll jelly (at 323 K), the correlation time $\tau_{i}$ is significantly (about five times) shorter than that for Haribo jelly and the coke, fish and pizza Vidal jelly. As far as the fast dynamics for Vidal roll jelly is concerned, the parameters of the dipolar relaxation constant yields $C_{f}^{DD}$ = 1.25 × 10^9^ Hz^2^, but the corresponding correlation time is also about five times shorter than for Haribo jelly and the coke, fish and pizza Vidal jelly group. Although, again, the assumption cannot be proven, it is expected that there are two pools of water molecules in jelly: a pool of confined (entrapped) water molecules, the dynamics of which are strongly restricted and slowed down by the macromolecular fraction, and a pool of water molecules, the dynamics of which are affected to a lesser extent by interactions with the macromolecular network. It is supposed that the fast dynamic process represents the movement of the second fraction of water molecules. The confined water molecules can perform complex motion, such as restricted translation diffusion (they can diffuse on the surface of the macromolecular network) or undergo rotational dynamics together with the macromolecules, mediated by exchange processes. One could think that the intermediate dynamics reflects the translation motion, while the slow dynamics represent the molecular tumbling. Eventually, for Vidal watermelon jelly, the dipolar relaxation constants, $C_{s}^{DD}$ and $C_{f}^{DD},$ have turned out to be lower (about five times for $C_{s}^{DD}$ and about 1.4 times for $C_{f}^{DD}$) than for Haribo jelly and the the coke, fish and pizza Vidal jelly group, while the correlation times match those for that jelly.

## 5. Materials and Methods

^1^H spin-lattice NMR relaxometry experiments have been performed for six kinds of Haribo jelly and six kinds of Vidal jelly. The experiments were carried out in a frequency range from about 10 kHz to 10 MHz at 298 K and 323 K, using a STELAR Smartracer relaxometer (STELAR, Mede, Italy). The relaxation process was single exponential for all kinds of jelly, in the whole frequency range at both temperatures. Examples of the ^1^H magnetization data recorded versus time are shown in the Supplementary Materials (Figures S1–S4). One should note that in some cases the relaxation processes are quickly compared to the switching time (3 ms). When the relaxation is fast, one detects in the experiment only the “tail” of the magnetization curve (magnetization versus time). This might influence the fitted relaxation rates. One should also note that the possibility of measuring short spin-lattice relaxation times depends on the spin–spin relaxation time that determines the length of the Free Induction Decay.

## 6. Conclusions

The extensive ^1^H spin-lattice relaxation studies for Haribo jelly and Vidal jelly show that NMR relaxometry gives a valuable insight into the dynamic and structural properties of jelly candies. Three dynamic processes contributing to the relaxation scenario have been identified; they can be referred to as slow (of the order of 10^−6^ s), intermediate (of the order of 10^−7^ s) and fast (of the order of 10^−8^ s) dynamics. It has been shown that the parameters characterizing the three dynamic processes in Haribo jelly are similar and this can be treated as a sign of their quality and authenticity. It has also been revealed that relaxation features of some Haribo jellies are affected by temperature, and increasing temperature mainly reduces the fraction of confined water molecules. 

The relaxation features of Vidal jelly at room temperature (298 K) are different than for Haribo jelly (in the low frequency range the relaxation process for Vidal jelly is too fast to determine the relaxation rates). At 323 K, two groups of Vidal jelly have been identified. For the first, including coke, fish and pizza Vidal jelly, the parameters (dipolar relaxation constants and correlation times) match those for Haribo jelly at 323 K. The second group includes cherry, roll and watermelon Vidal jelly. This group of Vidal jellies considerably differs from Haribo jelly and the coke, fish and pizza Vidal jelly in terms of the parameters (dipolar relaxation constants and correlation times) characterizing their dynamic properties.

## Figures and Tables

**Table 1:** Parameters characterizing molecular dynamics in Haribo jellies. $C_{s}^{DD}$ = 1.46 × 108 (± 6.1 × 10^7^) Hz^2^, $C_{f}^{DD}$ = 1.25 × 10^9^ (± 3.2 × 10^8^) Hz^2^ and $\tau_{f}$ = 5.21 × 10^−8^ (± 2.7 × 10^−9^) s for all cases, except for Haribo balla raspberry jelly at 323 K, in this case $C_{f}^{DD}$ = 1.02 × 10^9^ (± 3.4 × 10^8^) Hz^2^; $\tau_{s}$ at 323 K has been fixed at 2.43 × 10^−6^ s.

Temp. (K)	$\tau_{s}$ (s)	$C_{i}^{DD\;}$ (Hz^2^)	$\tau_{i}$ (s)	A (s^−1^)
Haribo bear jelly and Haribo phantasia jelly				
298	2.43 × 10^−6^ (± 5.2 × 10^−7^)	1.00 × 10^9^ (± 1.2 × 10^7^)	4.83 × 10^−7^ (± 3.9 × 10^−8^)	27.0 (±3.2)
323	2.43 × 10^−6^	8.02 × 10^8^ (± 4.3 × 10^7^)	3.49 × 10^−7^ (± 2.6 × 10^−8^)	27.0
Haribo balla apple jelly				
298	1.64 × 10^−6^ (± 6.9 × 10^−8^)	5.20 × 10^8^ (± 3.3 × 10^7^)	3.97 × 10^−7^ (± 2.9 × 10^−8^)	40.6 (±5.3)
323	1.50 × 10^−6^ (± 5.7 × 10^−8^)	4.12 × 10^8^ (± 2.9 × 10^7^)	3.00 × 10^−7^ (± 2.8 × 10^−8^)	40.6
Haribo balla raspberry jelly				
298	1.76 × 10^−6^ (± 1.3 × 10^−7^)	7.86 × 10^8^ (± 3.9 × 10^7^)	4.34 × 10^−7^ (± 3.0 × 10^−8^)	37.8 (±3.3)
323	1.37 × 10^−6^ (± 3.6 × 10^−8^)	1.78 × 10^8^ (± 2.1 × 10^7^)	2.74 × 10^−7^ (± 4.2 × 10^−8^)	37.8
Haribo tropifruity jelly and Haribo color-rado jelly				
298	1.92 × 10^−6^ (± 5.0 × 10^−8^)	7.08 × 10^8^ (± 1.6 × 10^7^)	4.17 × 10^−7^ (± 1.3 × 10^−8^)	33.1 (±3.7)
323	1.20 × 10^−6^ (± 5.8 × 10^−8^)	3.61 × 10^8^ (± 2.2 × 10^7^)	3.66 × 10^−7^ (± 3.3 × 10^−8^)	33.1

**Table 2:** Parameters characterizing molecular dynamics in Vidal jelly at 323 K. (In case there are no parameter uncertainties provided, the parameters have been set to the value given in the table and fixed).

$C_{s}^{DD}$ (Hz^2^)	$\tau_{s}$ (s)	$C_{i}^{DD}$ (Hz^2^)	$\tau_{i}$ (s)	$C_{f}^{DD\;}$ (Hz^2^)	$\tau_{f}$ [s]	A [s^−1^]
Vidal coke jelly, Vidal fish jelly and Vidal pizza jelly						
1.46 × 10^8^	1.53 × 10^−6^ (± 2.8 × 10^−8^)	6.98 × 10^8^ (± 6.9 × 10^7^)	2.53 × 10^−7^ (± 1.6 × 10^−8^)	1.25 × 10^9^	3.55 × 10^−8^ (± 6.3 × 10^−9^)	25.6 (±1.2)
Vidal cherry jelly						
1.16 × 10^7^ (± 3.1 × 10^6^)	2.55 × 10^−6^ (± 5.3 × 10^−7^)	1.38 × 10^8^ (± 1.7 × 10^7^)	3.45 × 10^−7^ (± 2.4 × 10^−8^)	9.11 × 10^8^ (± 3.1 × 10^7^)	5.61 × 10^−8^ (± 2.4 × 10^−9^)	38.4 (±2.2)
Vidal roll jelly						
1.46 × 10^8^	1.26 × 10^−6^ (± 3.0 × 10^−8^)	4.90 × 10^8^ (± 2.0 × 10^8^)	9.32 × 10^−8^ (± 2.6 × 10^−8^)	1.25 × 10^9^	9.68 × 10^−9^ (± 6.1 × 10^−9^)	15.1 (±0.8)
Vidal watermelon jelly						
2.94 × 10^7^ (± 8.8 × 10^7^)	2.44 × 10^−6^ (± 4.7 × 10^−7^)	1.64 × 10^8^ (± 2.5 × 10^7^)	4.27 × 10^−7^ (± 8.8 × 10^−8^)	8.79 × 10^8^ (± 6.6 × 10^7^)	4.39 × 10^−8^ (± 6.7 × 10^−9^)	35.0 (±3.3)

## Data Availability

https://zenodo.org/record/6992787.

## Supplementary Materials

The following supporting information can be downloaded at: https://www.mdpi.com/article/10.3390/molecules28052230/s1, Figure S1: Examples of ^1^H magnetization curves for different kinds of Haribo jelly at 298 K; Figure S2: Example of ^1^H magnetization curves for different kinds of Vidal jelly at 323 K; Figure S3: Example of ^1^H magnetization curves for different kinds of Haribo jelly at 298 K; Figure S4: Example of 1H magnetization curves for different kinds of Vidal jelly at 323 K.

## Appendix A

^1^H NMR relaxometry data for selected jelly interpreted in terms of the model described in theory.
