# Supplementary material for: Molecular Dynamics of Jelly Candies by Means of Nuclear Magnetic Resonance Relaxometry
Source: Molecules. 2023 Feb 27;28(5):2230. doi: 10.3390/molecules28052230 (PMC10005792; doi:10.3390/molecules28052230)
Supplement: Supplementary file 1 [file molecules-28-02230-s001.zip › molecules-1926387-supplementary.pdf]

Supplementary Materials

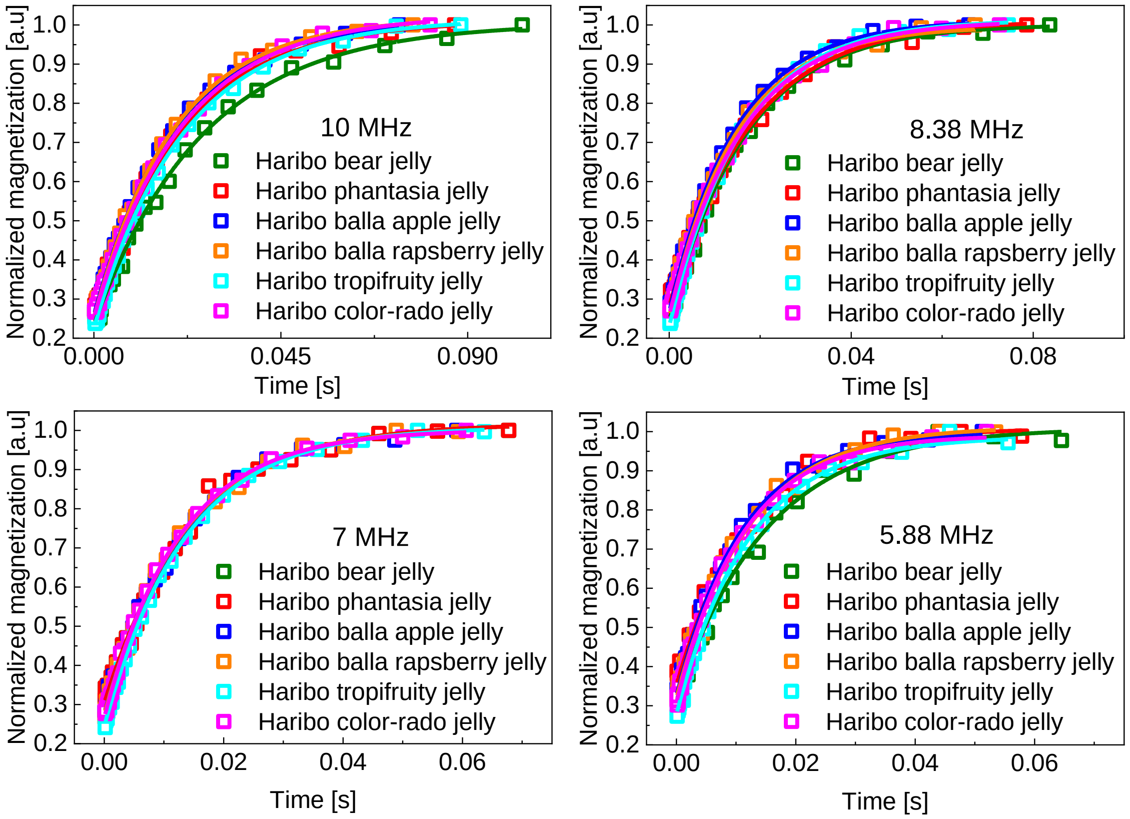

Figure. S1. Example of 1H magnetisation curves for different kinds of Haribo jelly at 298◦K; solid lines – single exponential fits.

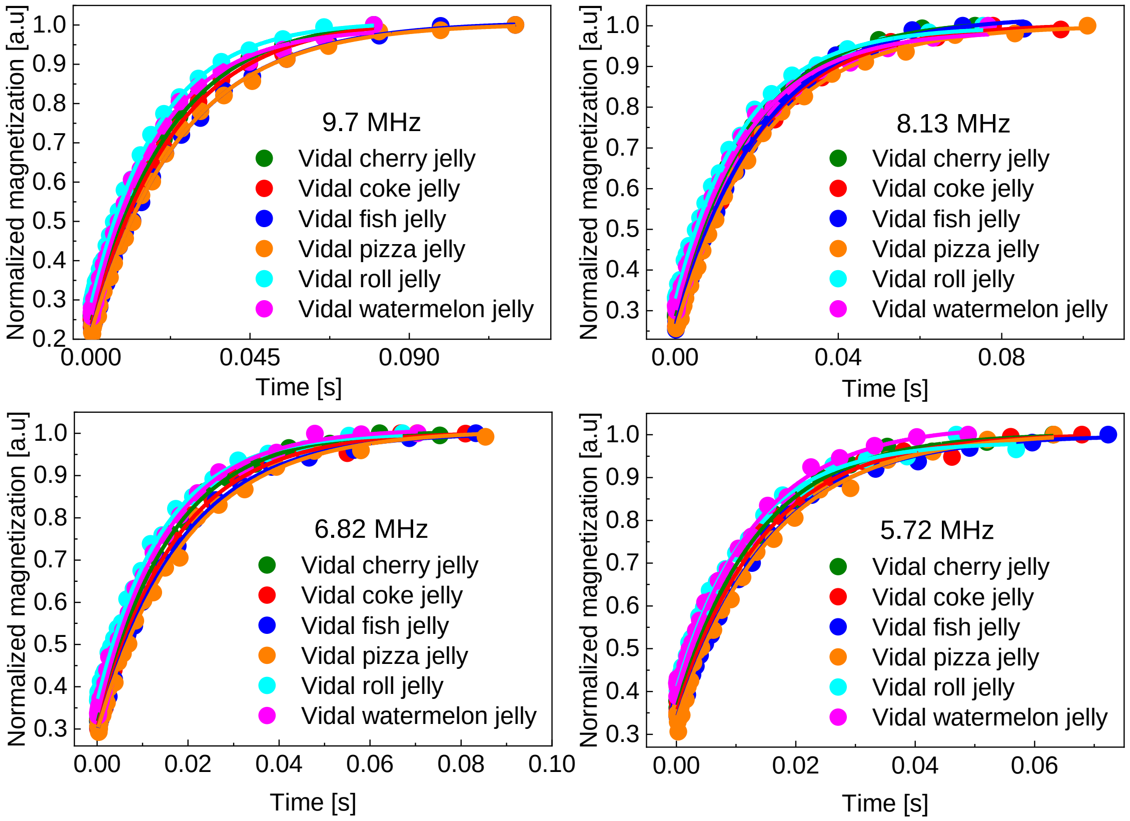

Figure. S2. Example of 1H magnetisation curves for different kinds of Vidal jelly at 323◦K; solid lines – single exponential fits.

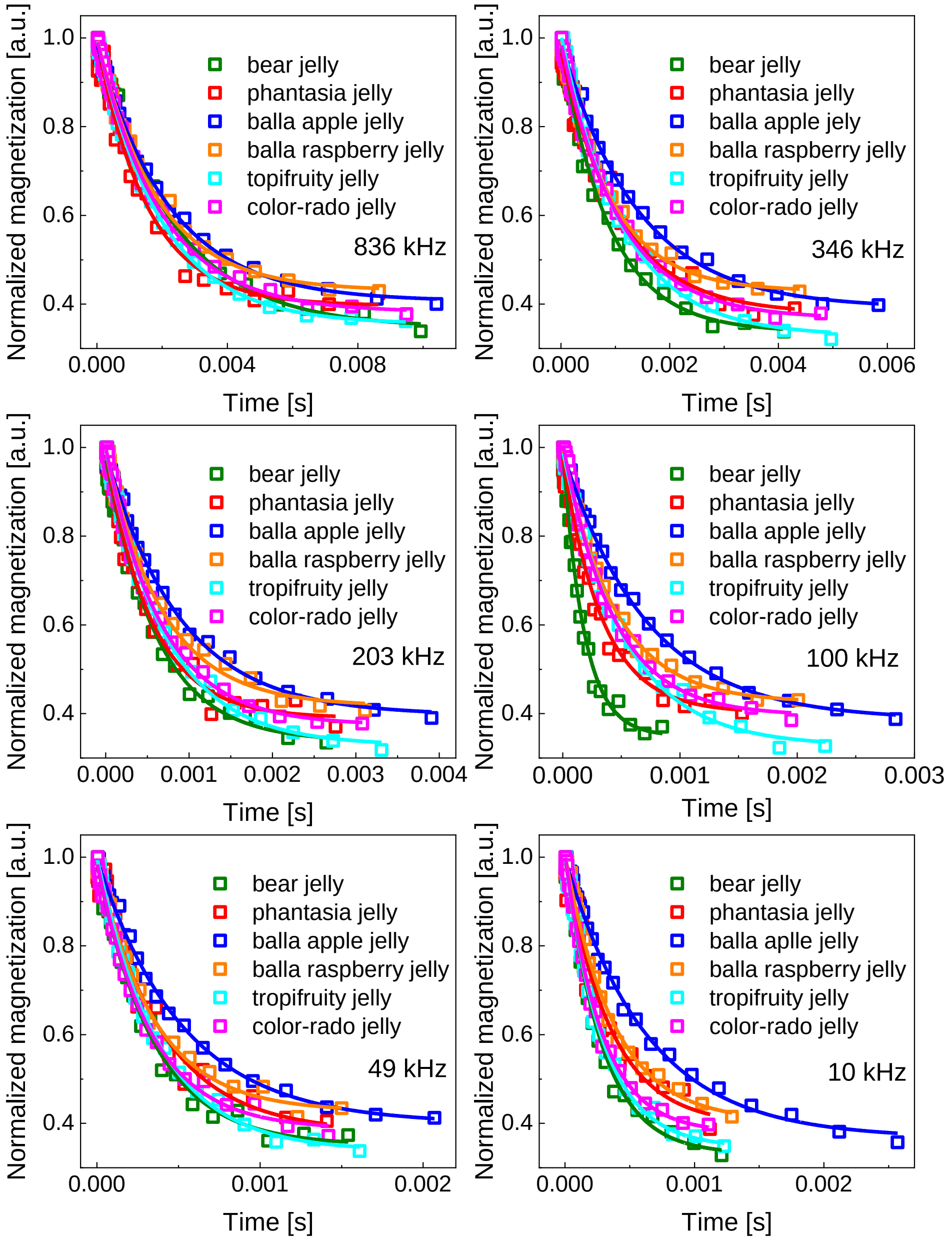

Figure S3. Example of 1H magnetisation curves for different kinds of Haribo jelly at 298◦K; solid lines – single exponential fits.

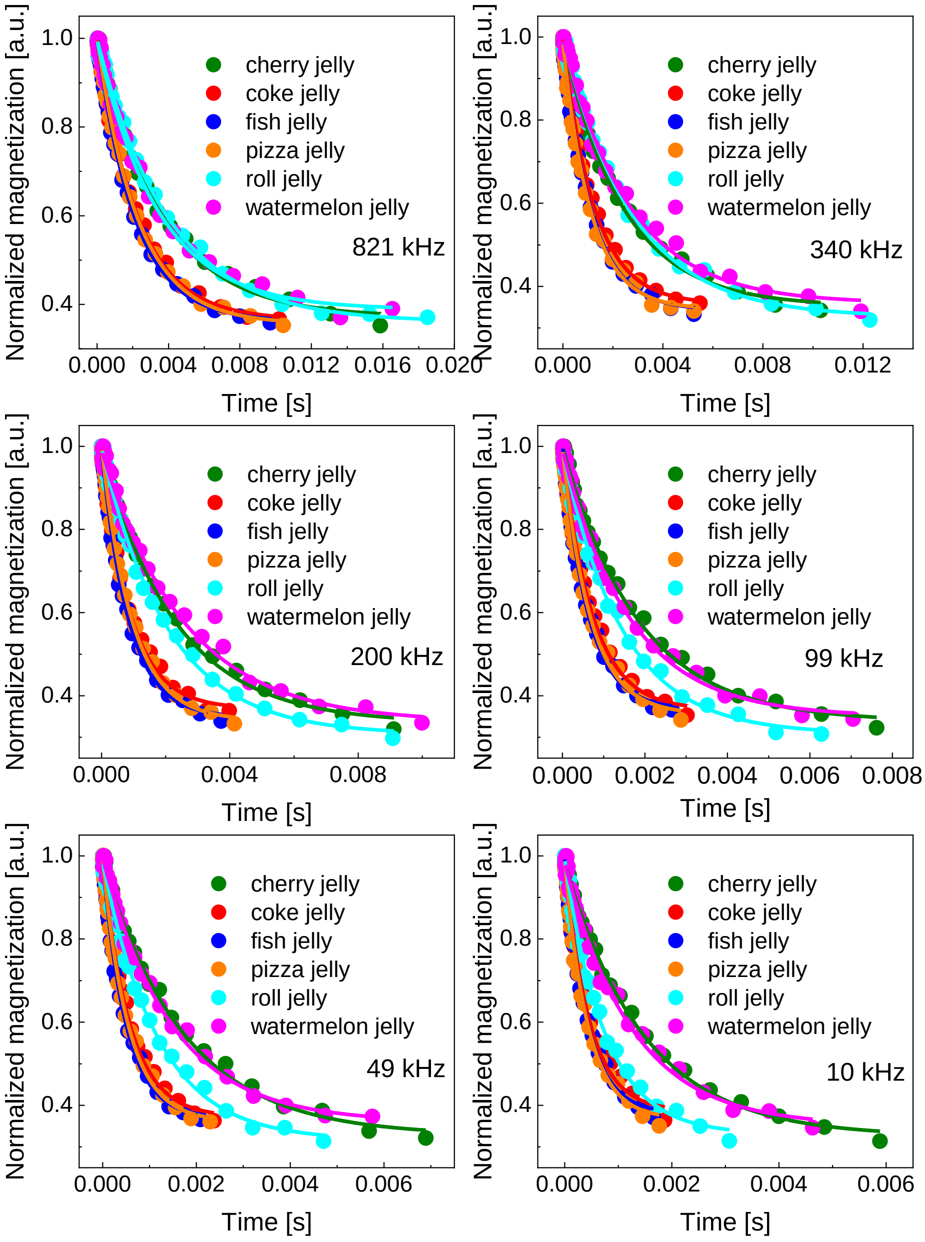

Figure S4. Example of 1H magnetisation curves for different kinds of Vidal jelly at 323◦K; solid lines – single exponential fits.
